# Predicting Depression Therapy Outcomes Using EEG-Derived Amplitude Polar Maps

**DOI:** 10.3390/brainsci15090977

**Published:** 2025-09-11

**Authors:** Hesam Akbari, Wael Korani, Sadiq Muhammad, Reza Rostami, Reza Kazemi, Muhammad Tariq Sadiq

**Affiliations:** 1 Department of Information Science, University of North Texas, Denton, TX 76205, USA; hesam.akbari@unt.edu; 2 Department of Data Science, University of North Texas, Denton, TX 76205, USA; wael.korani@unt.edu; 3 School of Computing, Gachon University, Seongnam-si 13120, Republic of Korea; tariqfast@gachon.ac.kr; 4 Department of Psychiatry, University of Tehran, Tehran 14155-6619, Iran; rrostami@ut.ac.ir (R.R.); reza.kazemi@ut.ac.ir (R.K.); 5 School of Computer Science and Electronic Engineering, University of Essex, Colchester CO4 3SQ, UK

**Keywords:** amplitude-polar map, feature engineering, SSRI, rTMS, depression therapy, EEG

## Abstract

**Background/Objectives**: Depression is a mental disorder that can lead to self-harm or suicidal thoughts if left untreated. Psychiatrists often face challenges in identifying the most effective courses of treatment for patients with depression. Two widely recommended depression-related therapies are selective serotonin reuptake inhibitors (SSRIs) and repetitive transcranial magnetic stimulation (rTMS). However, their response rates are approximately 50%, which is relatively low. This study introduces a computer-aided decision (CAD) system designed to determine the effectiveness of depression therapies and recommends the most appropriate treatments for patients. **Methods**: Each channel of the EEG is plotted in two-dimensional (2D) space via a novel technique called the amplitude polar map (APM). In each channel, the 2D plot of APM is utilized to extract distinctive features via the binary pattern of five successive lines method. The extracted features from each channel are fused to generalize the pattern of EEG signals. The most relevant features are selected via the neighborhood component analysis algorithm. The chosen features are input into a simple feed-forward neural network architecture to classify the EEG signal of a depressed patient into either a respondent to depression therapies or not. The 10-fold cross-validation strategy is employed to ensure unbiased results. **Results**: The results of our proposed CAD system show accuracy rates of 98.06% and 97.19% for predicting the outcomes of SSRI and rTMS therapies, respectively. In SSRI predictions, prefrontal and parietal channels such as F7, Fz, Fp2, P4, and Pz were the most informative, reflecting brain regions involved in emotional regulation and executive function. In contrast, rTMS prediction relied more on frontal, temporal, and occipital channels such as F4, O2, T5, T3, Cz, and T6, indicating broader network modulation via neuromodulation. **Conclusions**: The proposed CAD framework holds considerable promise as a clinical decision-support tool, assisting mental health professionals in identifying the most suitable therapeutic interventions for individuals with depression.

## 1. Introduction

According to the World Health Organization (WHO), depression is one of the most common mental disorders worldwide [1]. It significantly influences both personal well-being and social functioning, highlighting the urgent need for effective detection and treatment strategies. Depression must be treated in a timely manner because it can cause self-harm or even suicide. The WHO reports that suicide ranks as the fourth most prevalent cause of mortality among teenagers and young adults globally [1]. Although depression is a major risk factor for suicide, effective treatment is essential not only to reduce suicidal ideation but also to improve overall quality of life and social functioning.

Treatment of depression is mitigated via selective serotonin reuptake inhibitors (SSRI) antidepressants or magnetic pulses such as repetitive transcranial magnetic stimulation (rTMS), depending on the level of depression. However, the success rates of these depression therapies are not guaranteed. The reason is that the response of a patient’s brain changes from one patient to another. This variability in brain response across individuals highlights the inherently nonlinear dynamics of the brain, which operates as a complex and unpredictable system. If a depression therapy fails for a depressed patient, there might be an increase in the suicide risk for that patient. Time is a significant factor in patients with depression. Proposing a robust and reliable method to predict the outcome of therapies for patients with depression before starting the treatment helps to enhance the quality of life of patients with depression.

One of the difficulties facing medical teams is deciding on the optimum level of a depression treatment plan. Psychiatrists use their experiences and a trial and error strategy to select the best depression therapy for every individual case. In medical terminology, a patient who gets well after therapy is called a responder (R) to therapy and, likewise, a non-responder (NR) is a patient who does not get well after the therapy.

Predicting depression in R and NR patients has been the subject of several studies. Blood tests [2], medical imaging [3], DNA [4], and socioeconomic background [5] are some of the modalities that form the basis of these studies. These days, one of the most widely used neuroimaging method in healthcare is EEG. Compared with other modalities, recording a patient’s EEG offers several advantages including real-time, cost-effectiveness, and widespread availability in hospitals and local clinics.

According to some studies, EEG signals may be utilized as biomarker to forecast how well patients respond to treatments. This inspired us to create a CAD system that would help mental health professionals decide on the optimal treatment plan for individuals with depression. In the literature, the results of depression treatment have been predicted using the study of EEG data. According to the methodology employed, earlier research may be divided into four groups: time-domain, frequency-domain, time–frequency-domain, and convolutional neural network (CNN)-based approaches.

In time-domain-based methods, the information is extracted from the EEG signals in the time domain, whereas in frequency-domain-based methods, the EEG signals are transferred into the frequency domain and then the frequency spectrum of the EEG signals is used to extract the information. Similarly, in time–frequency-domain-based methods, the information is extracted from the EEG signals after decomposing the EEG signals into subbands. On the other hand, we have CNN-based methods in which the information is extracted from the EEG signals via successive filtering and max pooling operators. In prior studies, most CNN-driven architectures designed to forecast treatment response in depressive disorders via image-based representations of EEG signals—generated through methods such as time-frequency analysis and connectivity estimation.

In the following, we review some remarkable studies in the literature that were conducted to predict the outcomes of depression therapies for patients via EEG signals.

Several studies have explored EEG-based prediction of treatment responses in depression by employing diverse signal processing and machine learning techniques. In the time-frequency domain, transformations such as STFT, EMD, and wavelet-based decomposition have been applied to extract relevant EEG subband features for classification, with some approaches have reported over 90% prediction accuracy for SSRI therapy responders [6]. Similarly, rTMS-related studies have analyzed EEG signals via EMD to derive intrinsic mode functions, from which entropy-based features such as permutation entropy were extracted. Notably, increased entropy values in specific IMFs (especially from the frontal region) were associated with positive treatment outcomes [7].

In other efforts focusing on nonlinear dynamics, researchers have computed measures such as spectral coherence, mutual information, and hemispheric power ratios to build predictive models for SSRI efficacy [8]. Feature selection methods such as the Fisher discriminant ratios were paired with probabilistic classifiers to achieve high classification performance. Additionally, studies using time-domain and entropy-based EEG features, often in combination with clinical variables, have shown promise. For instance, microstate analysis, multiscale entropy, and spectral patterns derived from both electrodes and sources domains were utilized alongside machine learning models such as SVMs to predict treatment outcomes in small clinical cohorts [9].

In a study examining EEG channels individually [10], the F8 channel was identified as particularly useful, achieving an accuracy of 80% in classifying patient responses to rTMS therapy. Another research effort utilized a time-domain method, which extracts both linear and nonlinear EEG signal features from individual channels. These features were then filtered through the minimal-redundancy–maximal-relevance approach for feature selection before classification with a K-nearest neighbors (KNN) algorithm. Using the F8 channel data, this method also achieves a classification accuracy of 80% in predicting rTMS therapy outcomes, with classifier validation carried out through leave-one-out cross-validation.

In this time-domain-based study [11], EEG connectivity features were analyzed in patients receiving rTMS, and machine learning models such as SVMs were applied to classify the responders. The initial results showed promising levels of sensitivity and specificity in a small cohort, a subsequent validation using a substantially larger sample size failed to confirm these outcomes. Specifically, theta-band connectivity, once considered to be a strong predictor of therapeutic response within the early stages of treatment, did not generalize across the expanded dataset.

In a study using a time-domain methodology [12], researchers applied a machine learning framework to predict responses to SSRI therapy among 51 patients diagnosed with depression. The study considered three distinct sets of data: demographic information, resting-state EEG signals, and source-localized current densities. Initially, irrelevant features were eliminated via a random forest based feature selection process, followed by dimensionality reduction with principal component analysis (PCA). The selected features were then classified using several machine learning classifiers. Among these, the random forest classifier achieved the highest accuracy, with an 88% success rate in predicting SSRI therapy outcomes.

Another study using frequency-domain EEG analysis [13] extracted both total and normalized spectral energy measures from different brain areas, particularly the posterior and anterior regions. Additionally, ratios between frequency bands, including those involving beta and alpha rhythms, were computed. These spectral features were used as inputs to a gradient boosting decision tree classifier to estimate SSRIs treatment response. Among all the extracted features, the low-frequency power observed at the occipital electrodes (O1 and O2) were found to be the most informative.

In one study applying a time-domain approach [14], researchers explored EEG functional connectivity by analyzing correlations within the alpha frequency band. These alpha spectral correlation measures demonstrated promising predictive power for patient responses to rTMS therapy. When these features were used as inputs for an ElasticNet machine learning algorithm, the model achieved an area under the curve (AUC) of 0.91, effectively predicting treatment outcomes. Another investigation [15] employed a different time-domain strategy by assessing the stability of EEG signals through frontal alpha asymmetry (FAA). This study indicated that FAA measures remained stable across repeated evaluations and were unaffected by the specific type of antidepressant therapy administered.

In a referenced study [16], a hybrid approach combining time-domain and frequency-domain analysis was used to extract diverse EEG signal characteristics. These included complexity measures such as Lempel-Ziv and Katz fractal dimensions, statistical and spectral features such as correlation dimension, power spectral density, and bispectrum-derived metrics, along with activity-based indicators from the frontal and prefrontal regions and their aggregated forms. To refine the input space, the minimal-redundancy–maximal-relevance (mRMR) algorithm was employed for feature selection. The resulting feature set was then evaluated via a K-nearest neighbors (KNN) classifier within a leave-one-out cross-validation framework. This methodology achieved an accuracy of 93.5%, sensitivity of 91.3%, and specificity of 91.3% in predicting therapeutic response to rTMS.

In one study [17], researchers introduced a polygenic EEG approach that utilized functional connectivity features derived through fast independent component analysis (ICA) to forecast patient responses to rTMS therapy. These connectivity metrics were subsequently classified using discriminant analysis (DA), resulting in an area under the curve (AUC) of 0.735.

A study conducted by Ivan et al. [18] utilized a frequency-domain framework to analyze EEG-derived indicators—namely paroxysmal activity, alpha peak frequency, and frontal alpha asymmetry (FAA)—to assess their predictive value in determining patient responsiveness to antidepressant drugs. The researchers inferred that leveraging EEG biomarkers previously correlated with depressive symptoms could aid in personalizing treatment strategies effectively.

Additionally, in a study using a time-domain-based approach [19], researchers developed a sparse EEG latent space regression (SELSER) model. This model employed EEG signatures as input features to predict therapeutic outcomes for depression. The performance of the SELSER model was assessed by calculating the relative mean square error (RMSE), achieving a value of 5.68 in predicting treatment outcomes.

An international trial known as iSPOT-D [20], which focused on optimizing therapeutic approaches for individuals with depression, indicated that the use of sertraline hydrochloride could help stabilize abnormalities in the EEG alpha-peak activity. This study reported a clinical improvement rate of around 74Another investigation [17] proposed a polygenic EEG-based approach utilizing fast independent component analysis (ICA) to derive functional connectivity measures. These connectivity measures were subsequently analyzed through discriminant analysis (DA), achieving an AUC value of 0.735 for predicting rTMS treatment outcomes.

Another investigation [21] utilized low-resolution brain electromagnetic tomography (LORETA) to assess functional brain connectivity and compute effective coherence across different cortical areas. The study examined how coherence metrics in the alpha and theta frequency ranges could predict responses to antidepressant treatment. Functional connectivity in the alpha band was examined between two key brain areas: the rostral portion of the anterior cingulate cortex (rACC) and the anterior region of the insular cortex (aINS). This analysis yielded a sensitivity of 82% and a specificity of 86%, highlights their predictive relevance for therapeutic outcomes.

In a CNN-based approach [22], connectivity-based images were created from EEG channels for distinct frequency bands, including delta, theta, alpha, and beta. These images were subsequently analyzed via four sequentially arranged pre-trained transfer learning (TL) models integrated with a bi-directional long short-term memory (Bi-LSTM) network. This methodology resulted in a high classification accuracy (ACC) of 98.33%.

Another CNN-related investigation [23] similarly generated EEG connectivity images, which were then input into a hybrid network combining the pre-trained Xception model with a Bio-LSTM architecture. A subject-wise validation approach, specifically a leave-one-out strategy, was employed. This method resulted in an impressive predictive accuracy of 98.86% for classifying responses to rTMS treatment.

In another convolutional neural network (CNN)-based investigation, Yerguzel et al. [24] evaluated the use of neural models by applying Cordance features extracted from EEG recordings captured during a resting-state protocol. The team implemented a relatively simple neural network consisting of 10 computational layers and achieved a classification accuracy of 89.09% for identifying responses to rTMS treatment.

In another CNN-focused research [25], EEG signals were transformed into images through continuous wavelet transform (CWT), which were then classified using an ensemble model built upon five pre-trained TL architectures. By employing a voting approach across these models, a classification accuracy of 96.55% was reported in predicting SSRI therapy outcomes.

Moreover, a different CNN approach [26] employed time-frequency representations of EEG data generated via CWT. These representations were analyzed by the EfficientNetB0-Bio-LSTM architecture, further enhanced with an attention mechanism. The method achieved a high accuracy rate of 97.1% for predicting rTMS therapy outcomes.

Another CNN-based model [27] took EEG signals as direct inputs without initial transformation. These raw signals were processed through a hybrid TL approach combined with a Bio-LSTM framework, reaching impressive classification accuracy of 98.84% in predicting patient responses to SSRI treatment.

In a deep learning-based approach [28], multiple transfer learning models—such as InceptionResNetV2-Bio-LSTM and its variants—were used to derive EEG features. These models were aggregated via a majority voting ensemble, where the influence of each model was fine-tuned via heuristic optimization techniques like differential evolution, resulting in classification accuracies exceeding 98% in some cases. A related study [28] transformed brainwave interactions into connectivity images, which were fed into a sequence of pre-trained convolutional networks including the EfficientNet, DenseNet, and VGG architectures. DenseNet and VGG models demonstrated strong baseline performance, and ensemble-based optimization further increased the predictive accuracy to over 92%. Summary of the studies in the literature is presented in Table 1.

EEG signals exhibit complex dynamic behaviors in high-dimensional space, making them effective in capturing the brain’s nonlinear characteristics. In this study, we avoid labeling the brain’s dynamics as strictly “chaotic” or “orderly.” Instead, we characterize variations in EEG activity using nonlinear dynamic features that reflect signal complexity over time.

However, only a limited number of studies have explored computer-aided systems specifically designed to predict treatment response in patients with depressive disorders. This is largely because of the computational demands involved in mapping brain signals into higher-dimensional feature representations. Consequently, identifying informative EEG patterns and reducing dimensionality becomes crucial. Sophisticated techniques for signal decomposition and selection are typically employed to isolate the most informative features for accurate classification [29,30].

**Table 1:** Summary of prior studies compared with the proposed method.

Study	Analytical Techniques and Models	Therapy	Sample Size (R vs. NR)	Accuracy	Sensitivity	Specificity
Shahabi et al., 2023 [28]	Transfer learning ensemble using Bio-LSTM	rTMS	23 vs. 23	98.51%	98.64%	98.36%
Khodayari-Rostamabad et al., 2013 [8]	Nonlinear EEG features + Fisher ratio + MFA classifier	SSRI	11 vs. 11	87.40%	94.90%	80.90%
Minami et al., 2022 [21]	Brain source connectivity and coherence + ROC analysis	SSRI	12 vs. 18	-	82.00%	86.00%
Jaworska et al., 2019 [12]	Demographic & EEG + PCA + RF classifier	SSRI	27 vs. 24	88.00%	77.00%	99.00%
Mumtaz et al., 2017 [6]	EEG time-frequency analysis + ROC curve + logistic regression	SSRI	16 vs. 18	91.60%	90.00%	90.00%
Shahabi et al., 2023 [26]	Time-frequency domain (CWT) + transfer learning + Bio-LSTM	rTMS	23 vs. 23	97.1%	97.3%	97.0%
Zhdanov et al., 2020 [9]	EEG + clinical variables + statistical filtering + SVM	SSRI	155 vs. 67	82.40%	72.90%	85.50%
Shahabi et al., 2022 [27]	Deep TL models fused with Bio-LSTM	SSRI	12 vs. 18	98.84%	97.80%	99.60%
Shahabi et al., 2023 [23]	Channel connectivity image + ensemble TL + LSTM	rTMS	23 vs. 23	99.32%	-	98.34%
Mirjebreili et al., 2024 [22]	EEG rhythms + channel connectivity + sequential TL + Bio-LSTM	SSRI	12 vs. 18	98.33%	-	-
Shahabi et al., 2021 [25]	Time-frequency plots using CWT + TL ensemble voting	SSRI	12 vs. 18	96.55%	96.01%	96.95%
Shahabi et al., 2023 [31]	Channel-wise connectivity features + ensemble TL models	rTMS	34 depressed	92.28%	-	-
**Proposed (Ours)**	APM-based 2D signal mapping + BPFSL feature encoding + NCA + FFNN	SSRI	12 vs. 18	98.06%	98.22%	97.97%
		rTMS	9 vs. 6	100%	100%	100%
		rTMS	23 vs. 23	97.19%	97.61%	96.80%

To avoid overinterpreting the electrophysiological basis of EEG dynamics, we have refrained from characterizing brain activity as “chaotic”. Instead, our framework treats EEG complexity as a reflection of underlying neurodynamic variability rather than assigning metaphysical descriptors.

However, there is not much studies in designing a CAD system to predict the outcomes of depression therapies. The reason for this limitation is posed by the computationally intensive steps of traditional techniques required to project signals onto higher-dimensional space.

In addition, extracting meaningful information from data is fundamental for identifying unique patterns and minimizing dimensionality for clearer data representation [29]. To enhance classification performance, both feature extraction methods and selection algorithms are employed to isolate the most informative attributes from the dataset [30]. Prior research on EEG-based prediction techniques can be broadly grouped into three categories: frequency-domain, time-domain, and time–frequency domain approaches.

Frequency-based methods often rely on spectral analysis tools such as the Fourier transform to quantify brain signal rhythms [8,13]. In contrast, time-frequency approaches analyze sub-band components via techniques such as wavelet transforms or empirical decomposition [6,25]. While these methods offer rich temporal and spectral insights, they tend to suffer from limitations such as high computational cost, difficulties with non-stationary signals, and sensitivity to noise or improper parameter selection—such as wavelet type and window size.

On the other hand, time-domain approaches directly extract statistical descriptors and structural patterns from the raw EEG signal, often using binary-based and determinant measures [8,9,12]. While models developed using such features typically achieve approximately 80% accuracy, these methods are simpler and more interpretable [8,9,12,13,17,19,21]. However, there remains a need to design time-domain strategies that can extract more refined and discriminative signal characteristics to enhance the predictive performance of machine learning models.

A critical analysis of existing research reveals several noteworthy shortcomings in prior studies:

- Traditional EEG features often fall short of 90% accuracy, mainly because they struggle to represent the brain’s inherently nonlinear characteristics.
- Clinicians lack intuitive visualization tools to interpret disordered neural patterns observed in depression, making real-time monitoring difficult.
- The majority of the previous deep learning studies are complex and demand significant computational resources.
- All previous studies proposed a single therapy rather than a model for multiple depression therapies.
- Previous studies have shown weaknesses in preprocessing EEG signals to enhance the performance of the proposed model.

For chaotic time series EEG signals, sine, and cosine functions are plotted against each other can reveal a strange attractor in a two-dimensional (2D) space, which is a key feature of chaotic systems. These attractors are usually fractal in nature, and their structure provides insights into the system’s dynamics. This fact inspired us to propose a method called the amplitude-polar map (APM) technique to plot the EEG signal in 2D space.

Furthermore, we introduce a new feature engineering method—Binary Pattern of Five Successive Lines (BPFSL)—which is derived from our APM approach. This technique is designed to capture the system’s nonlinear behavior and complexity while retaining its immediate dynamic characteristics. We then fuse resulting features of BPFSL of all EEG channels. This fusion process results in a generalized pattern that captures the nonlinear relationships between the EEG signal channels and the instantaneous dynamics of multi-channel EEG data.

This work presents a CAD system to predict the best course of depression therapy. The multi-channel EEG signals are de-noised via a multi-scale principal component analysis (MSPCA), and the EEG signals are plotted in 2D space via APM technique. The nonlinearity and complex behavior of the EEG shapes in 2D space are utilized to extract distinctive features via our proposed BPFSL technique. The Neighborhood Component Analysis (NCA) is implemented to select the best group of features. These features are used to propose a classifier via a feedforward neural network (FFNN) with a 10-fold CV strategy. Two binary classification tasks are defined: R to SSRI vs. NR to SSRI and R to rTMS vs. NR to rTMS. Our contributions to predict the best course for depression therapy are represented as follows:

- We propose a novel technique to represent the complexity of EEG signals in a 2D space.
- We propose a novel feature engineering technique in the time domain on the basis of the APM shape on 2D space, called BPFSL, to extract distinctive features of EEG signals.
- Design and validation of a reliable CAD system for recommending the best course of depression therapy.
- Compared with the previous model, we proposed the high-performance model in terms of classification ACC.
- Deliver a computationally efficient CAD system.
- We integrate the proposed CAD system into software to support psychiatrists’ decisions.

The present work is structured as follows: Section 2 describes the materials. Section 3 describes the pre-prossessing of databases. The proposed CAD system is explained in Section 4. Results and discussion are reported in Section 5. The paper is concluded in Section 6.

## 2. Materials

In this research, the effectiveness of the developed CAD system in recommending optimal depression treatment options are assessed via two distinct EEG datasets. Specifically, the evaluation involves analyzing the performance of the CAD system for predicting patient responses to SSRI and rTMS therapies. The datasets utilized include a publicly available dataset, termed here as the Mumtaz dataset, and a privately collected dataset obtained from Atieh Hospital, referred to as the Atieh Hospital dataset. The following section outlines comprehensive details about these two datasets.

### 2.1. Mumtaz Database

In this study, we employed the publicly available Mumtaz EEG dataset to evaluate the developed model’s effectiveness in predicting patient responses to SSRI treatment [32]. This dataset is accessible via Figshare (https://figshare.com/articles/dataset/EEG_Data_New/4244171 (accessed on 1 January 2025)) and includes EEG recordings collected from 30 patients diagnosed with major depressive disorder (MDD) according to the Diagnostic and Statistical Manual-IV (DSM-IV) criteria.

All patients included in the dataset were about to start SSRI medication, and EEG data were captured beforehand using the standard 10–20 international EEG electrode system. EEG signals were sampled at 256 Hz and recorded for approximately five minutes while patients rested quietly with their eyes closed.

Patients undergoing SSRI therapy were prescribed medications such as escitalopram, sertraline, fluoxetine, or paroxetine. These medications were administered as part of the clinical treatment regimen and were not altered for research purposes.

Each patient received a specified dosage of SSRI medication for a period of four weeks. Post-treatment assessment involved the use of the Beck Depression Inventory (BDI) scores, with patients exhibiting more than a 50% improvement compared with baseline classified as responders (R), and those showing less improvement were categorized as non-responders (NR). Following the four-week treatment, 12 out of the 30 patients were classified as responders, whereas the remaining 18 showed insufficient improvement and were labeled as non-responders.

**Figure 1:**
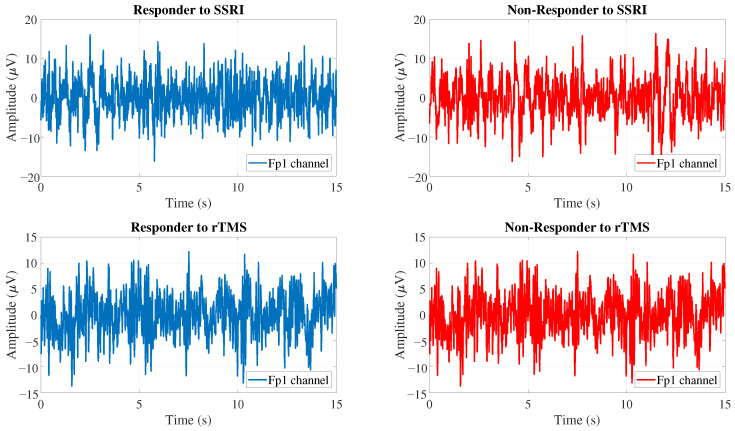
EEG samples from responders and non-responders to SSRI and rTMS therapies.

This research aims to establish an effective classification model to predict SSRI therapy outcomes by analyzing EEG data acquired prior to treatment initiation. Demographic characteristics for the participants included in the Mumtaz database are detailed in Table 2. For analysis purposes, EEG recordings from this database were segmented into intervals of 15 s. Dataset comprises signals from 19 EEG channels. Examples of EEG signals corresponding to both R and NR patient groups are illustrated in Figure 1.

### 2.2. Atieh Hospital Database

To validate the CAD system for rTMS treatment prediction, we used a proprietary EEG dataset collected at Atieh Hospital in Tehran, Iran. Diagnoses of depression were made by certified clinical professionals, and EEG signals were obtained prior to therapy using a 19-channel configuration following the 10–20 system at a 500 Hz sampling rate. Patients were evaluated via the Beck Depression Inventory (BDI) both before and four weeks after receiving rTMS. Individuals who showed a reduction of 50% or more in BDI scores were considered responders, whereas the others were labeled non-responders. The outcome classification was cross-verified by mental health specialists to ensure clinical reliability.

In the rTMS group, all patients were concurrently taking antidepressants as part of their ongoing treatment plan. However, the Atieh Hospital dataset does not consistently document the names or dosages of these medications, so specific drug details could not be reported.

The Atieh Hospital database consists of two subsets: a small subset and a large subset. The small subset includes EEG recordings from 15 patients diagnosed with depression, comprising 9 responders (R) and 6 non-responders (NR), while the large subset includes recordings from 46 patients—23 R and 23 NR. The summary of BDI-II scores before and after rTMS therapy for the small subset is presented in Table 3, and detailed clinical and demographic characteristics for the large subset are reported in Table 4. Notably, demographic details such as age, gender distribution, and duration of depression were not provided for the small subset, but were available and statistically analyzed for the large dataset.

EEG signals in both datasets were recorded from 19 electrodes using the international 10–20 system and segmented into 15-s epochs for analysis. An illustrative comparison of EEG samples for R and NR groups is provided in Figure 1.

In this study, the rTMS therapy protocol followed the standard high-frequency stimulation method approved for treating major depressive disorder. Each patient received 10 Hz stimulation over the left dorsolateral prefrontal cortex (DLPFC), with a total of 3000 pulses per session. Sessions were conducted five times a week for four consecutive weeks, resulting in a total of 20 sessions. The stimulation intensity was set at 110% of the patient’s resting motor threshold. Clinical evaluations using the BDI scale were conducted before the start of therapy and after the final session. The therapy was administered under the supervision of a clinical psychiatrist and trained technician.

## 3. Pre-Prossessing

The power-line noise and motion artifacts of the EEG signals of the Mumtaz and Atieh Hospital databases are removed via EEGLAB software (version 2024.2.1, UC San Diego, CA, USA) [33]. The EEG signals are filtered by a high-pass finite impulse response (FIR) filter and a low-pass filter at 0.5 Hz and 45 Hz, respectively. Additionally, a notch filter (band-stop filter) with a bandwidth of 47–53 Hz was applied to eliminate power-line noise.

EEG signals were divided into 15-s intervals. After segmentation, the Mumtaz dataset yielded 244 and 344 segments labelled responders (R) and non-responders (NR) for SSRI treatment. A similar segmentation procedure was performed at Atieh Hospital datasets. Within the smaller subset of the Atieh Hospital data, 182 and 112 EEG segments were identified as R and NR for rTMS therapy. In the larger Atieh Hospital dataset, 437 segments were labeled as responders and 419 as non-responders, also for rTMS-based analysis.

## 4. Methods

**Figure 2:**
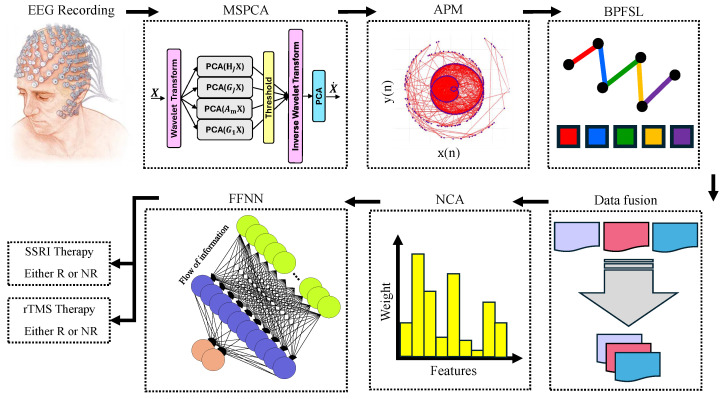
Workflow of the proposed CAD framework for forecasting depression treatment response. EEG signals are first recorded and denoised using Multiscale PCA (MSPCA). Amplitude Phase Maps (APM) are generated and input to the BP Feature Selection Layer (BP-FSL) to extract key patterns. These features are fused and passed through Neighborhood Component Analysis (NCA) for dimensionality reduction and feature weighting. A Feedforward Neural Network (FFNN) is then used to classify patients as Responders (R) or Non-Responders (NR) to either SSRI or rTMS therapy.

This section outlines the sequence of operations involved in the proposed CAD framework developed for forecasting patient responses to depression treatment. A visual summary of the system’s workflow is provided in Figure 2.

### 4.1. Multiscale Principal Component Analysis

**Figure 3:**
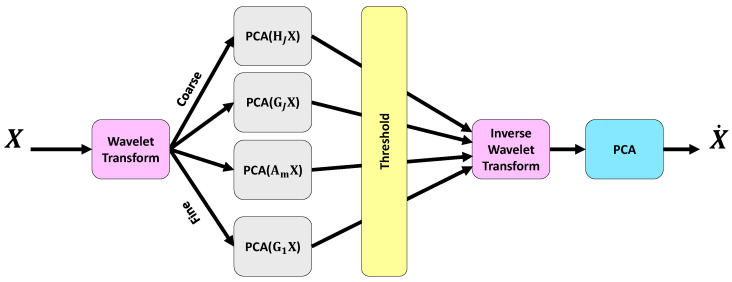
Illustration of the MSPCA methodology used for EEG denoising. The input EEG signal *X* is decomposed into coarse and fine components using a wavelet transform. Principal Component Analysis (PCA) is applied separately to each component to suppress noise while preserving signal structure. After thresholding, an inverse wavelet transform reconstructs the signal, followed by a final PCA step to obtain the denoised output $\dot{X}$.

Although EEG signals are passed through 3 flitters as the initial filtering in pre-processing, there are still various noise artifacts such as systematic, blink, and thermal noises. To remove these artifacts, the multi-scale principal component analysis (MSPCA) algorithm is applied to the EEG signals as illustrated in Figure 3. The noisy EEG signals can be modelled linearly as

(1) $$X=X_{EEG}+X_{N}$$

where $X_{EEG}$ represents the clean multichannel EEG signal, and $X_{N}$ denotes the noise component.

The goal of noise reduction techniques is to remove $X_{N}$ from $X_{EEG}$. PCA is a commonly employed technique for noise cancelling. In fact, the PCA reduces the correlation among variables by establishing a linear relationship between observations [34,35]. Since EEG signals are inherently non-stationary and nonlinear, wavelet transform techniques are also utilized for effective signal analysis. By combining the strengths of PCA and wavelet transform, the MSPCA approach is developed. The steps of MSPCA is depicted in Figure 3. The steps of the MSPCA technique are as follows [34,36]:

1. Decompose each column of a given data matrix *X* via wavelet transform up to a specified level *J*.
2. For 1≤m≤J, apply PCA to the detailed coefficients $G_{m}X$, retaining only the principal components with eigenvalues exceeding the average eigenvalue, as determined by the Kaiser rule [34], or discard the details otherwise.
3. PCA is performed on the approximation coefficients $H_{J}X$, retaining the principal components whose eigenvalues exceed the average eigenvalue, again on the basis of the Kaiser rule [34].
4. Reconstructing a new matrix from the reduced detail and approximation coefficients using the inverse wavelet transform.
5. PCA is applied to the reconstructed matrix to obtain the final processed matrix $\dot{X}$.

In the current work, the MSPCA algorithm is applied to multichannel EEG signals for efficient noise cancellation. The MSPCA is implemented via the *wmulden* function on the MATLAB software (R2024a, MathWorks, Natick, MA, USA).

### 4.2. Amplitude-Polar Map

Chaos theory is used to analyze nonlinear and complex systems, such as the brain. Time series generated by chaotic systems, such as EEG signals from the brain, are complex and exhibit intricate behavior. Phase space reconstruction (PSR) is one of the most common used techniques in chaos theory for decoding the dynamic behavior of complex and nonlinear time series by mapping them into a higher-dimensional space [37]. The PSR technique requires selecting parameters such as the embedding dimension and time delay to accurately capture the complexity and dynamics of a time series. Choosing these parameters of PSR is challenging: setting them too high causes the PSR shape to become unnecessarily complex, while setting them too low prevents the PSR shape from adequately representing the nature complexity of the time series [37].

The second-order difference plot (SODP) is another technique used to illustrate the EEG signals in 2D space [38]. The SODP shows the amplitude variations in EEG signals. However, its limitation is that it reflects only changes in the amplitude of EEG signals. It provides no information about the complex behaviors of the EEG signals [38].

The complex plot (CP) is another method for representing the complexity of an EEG signals on the 2D space [39]. The major drawback of the CP is that it requires the quantification of the amplitude changes of EEG signals before plotting, leading to the loss of valuable information about the time-domain dynamics of the signals [39].

For a chaotic time series, its sine and cosine functions are plotted against each other can reveal a strange attractor in a 2D space which is a key feature of chaotic systems. These attractors are typically fractal in nature, with their structure offering insights into the system dynamics. Inspired by this concept, we propose the amplitude-polar map (APM) technique to visualize the behavior EEG signals in a 2D space.

The APM plots the sine and cosine of EEG signals aligned with the variation of the EEG signal in a 2D space instead of performing traditional techniques such as PSR, SODP and CP [37,38,39]. The APM technique provides a more stable and robust plot for analyzing the dynamics of EEG signals by simplifying the complexity of the time series while preserving essential information about complex behavior. For a complex time series such as an EEG signal, representing a 2D plot via sine and cosine functions is easier and faster than traditional techniques do. The APM is described as follows.

**Figure 4:**
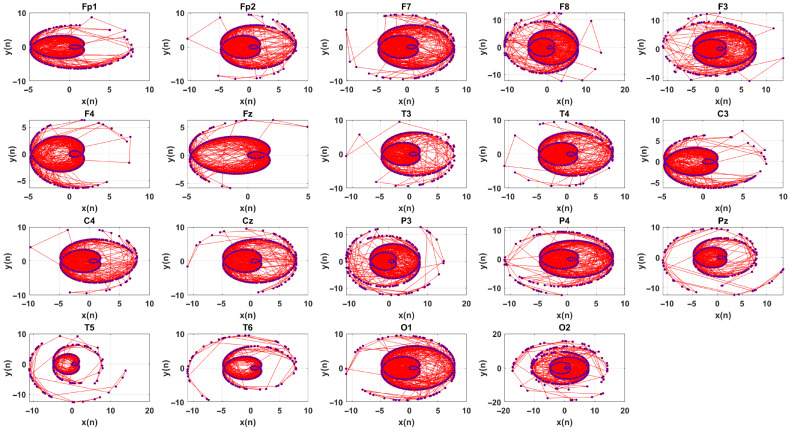
APM of a 19 channel EEG signal.

Assume that a(n) is one channel of the EEG signal with *N* samples. Its differential is computed as follows:

(2) $$a^{\prime }\left(n\right)=a\left(n\right)-a\left(n-1\right)$$

where $a^{\prime }\left(n\right),a\left(n\right)$ and a(n−1) are the first-order differentials at sample *n*, and the value of EEG signal at sample *n*, the value of the EEG signal at sample n−1, respectively. Then x(n) and y(n) are defined as follows:

(3) $$x\left(n\right)=a^{\prime }\left(n\right)\times sin\left(a^{\prime }\left(n\right)\right)$$

(4) $$y\left(n\right)=a^{\prime }\left(n\right)\times cos\left(a^{\prime }\left(n\right)\right)$$

the APM is then defined by plotting x(n) vs. y(n). The APM technique maps the amplitude-dependent oscillations of the EEG signal in a 2D space. The APM reveals geometric complexity and dynamic behavior of EEG signals. Dense regions indicate slow variations, while spread regions highlight rapid fluctuations or nonlinear behaviors. Figure 4 shows APMs of the EEG signal.

### 4.3. Binary Pattern of Five Successive Lines

Several features have been defined in the literature to quantify the nonlinearity of a 2D shape in higher-dimensional spaces, such as the fractal dimension, the largest Lyapunov exponent, and the correlation dimension. These features characterize the system complexity by analyzing the trajectory of its states after plotting the 2D shape. Fractal dimension quantifies the self-similarity of the time series. The Lyapunov exponent evaluates the predictability of the time series by measuring the rate of divergence of nearby trajectories. The correlation dimension measures the statistical self-similarity of the system to assess the complexity of the time series.

These traditional features cannot fully capture the complex and true underlying dynamics of a multi time series, such as multichannel EEG signals. Another disadvantage of these traditional features is their sensitivity to noise and the inherent difficulty in accurately reconstructing the PSR shape from real-world data, such as multi-channel EEG signals.

**Figure 5:**
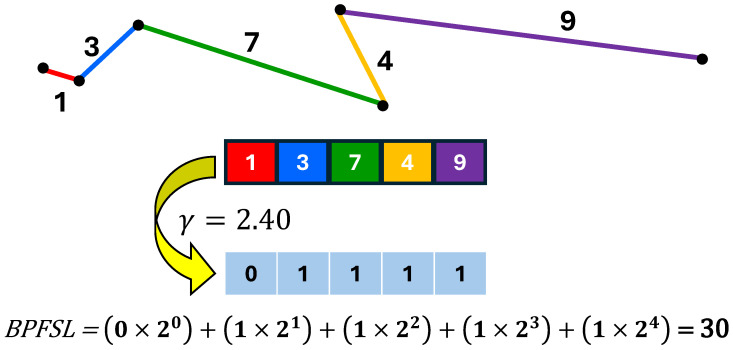
Illustration of the BPFSL technique with an example.

The variation in the 2D shape might be used as a significant parameter to capture the dynamics of the data. This inspired us to compute the binary pattern of five successive lines (BPFSL) to decode the nonlinearity of the EEG signals in 2D space. We defined plotting x(n) vs. y(n) as the APM in Section 4.2. The distance between two successive points in the APM plot is calculated as follows:

(5) $$A\left(n\right)=\sqrt{{\left(x\left(n+1\right)-x\left(n\right)\right)}^{2}+{\left(y\left(n+1\right)-y\left(n\right)\right)}^{2}}$$

six successive points make five successive lines and variation of these successive five lines can be quantized by five bits as

(6) $$BPFSL\left(n\right)=\sum_{i=0}^{4}f\left(A\left(n+i\right),\gamma \left(n\right)\right)\times 2^{i}$$

where γ(n) and f(.,.) are defined as follows:

(7) $$\gamma \left(n\right)=\frac{\mathrm{standard}\mathrm{deviation}(A(n),A(n+1),A(n+2),A(n+3),A(n+4))}{\sqrt[{4}]{2}}$$

(8) $$f\left(\alpha ,\beta \right)=\left\{\begin{array}{cc} 1 & \mathrm{if}\;\alpha \geq \beta \\ 0 & \mathrm{if}\;\alpha <\beta \end{array}\right.$$

then, we utilize as histogram to decode the pattern of BPFSL(n) as follows:

(9) $$H\left(BPFSL\left(n\right)\right)=\left[h_{1},h_{2},\ldots ,h_{i}\right]$$

where *i* is the number of bins, and $h_{i}$ represents the frequency of occurrence. Each histogram bin contributes a single feature, making the number of features equivalent to the number of bins. Since five bits are used to quantize the BPFSL(n), its range becomes between 1 and $2^{5}=32$. Therefore, we set the number of bins for the histogram to 32 (i.e., i=32). Figure 5 shows an example of the BPFSL technique.

Uniqueness of the BPFSL Method: Unlike conventional feature extraction approaches that treat EEG features independently, the proposed BPFSL method is specifically designed to exploit spatial and hemispheric symmetry inherent in EEG polar maps. By using a five-line binary encoding mechanism, BPFSL captures nonlinear spatial dynamics and inter-hemispheric correlations in a compact form. It processes left and right hemisphere information in parallel via dual-stream feature encoding, which enhances robustness when distinguishing subtle variations between responders and non-responders. This makes BPFSL particularly suited to clinical applications where subtle EEG variations are clinically meaningful.

### 4.4. Data Fusion

**Figure 6:**
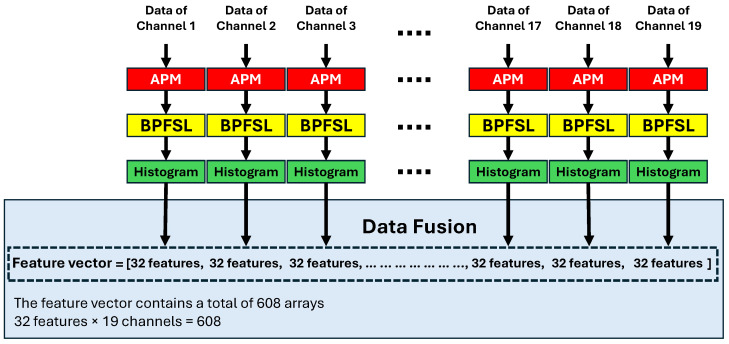
Illustration of data fusion.

Each channel of the EEG signal is plotted in 2D space via the APM technique. Then, the BPFSL is applied to each individual channel, and 32 features are extracted. Since there are 19 channels in the EEG signals, and the total number of features, 19×32=608, are extracted via concatenating the features of all channels. This can be defined mathematically as follows:

(10) $$F=[H_{Ch_{1}},H_{Ch_{2}},\ldots ,H_{Ch_{19}}]$$

where *F* is the final feature vector, and $H_{Ch_{i}}$ represents the 32 features extracted from the *i*th channel. Fusing the features from each EEG channel by concatenation provides a comprehensive representation of the brain’s activity across all channels. The resulting fused vectors, obtained by concatenating features from multiple channels, offer a more generalized patterns that can improve the performance of machine learning models, leading to an increase in CAD system performance. Additionally, the fusion of features by concatenation preserves channel-specific information while enabling the exploration of relationships between signals from different brain regions. An illustration of this process is shown in Figure 6.

### 4.5. Neighborhood Component Analysis

Neighborhood Component Analysis (NCA) is a supervised feature selection technique that optimizes the performance of nearest neighbor classifiers by learning a feature weighting matrix. NCA transforms the feature space in such a way that data points from the same class are brought closer together, which increases the classification accuracy. NCA uses gradient-based optimization to determine the importance of each feature, discarding irrelevant and redundant ones. In other words, NCA performs dimensionality reduction while capturing nonlinear feature interactions. In the current work, NCA is used to rank the features on the basis of their weights. More details on the NCA methodology can be found in [40]. For feature selection, the *fscnca* MATLAB function is used to implement the NCA algorithm.

### 4.6. Feedforward Neural Network

Selecting an appropriate neural network architecture is crucial for enhancing the predictive accuracy of a CAD system, particularly when dealing with previously unseen data. In this framework, a basic form of artificial neural network known as a Feedforward Neural Network (FFNN) is utilized. This model includes one hidden layer, making it a relatively shallow structure. It is employed to separate input features into two groups: responders (R) and non responders (NR).

In a feedforward neural network (FFNN), information progresses sequentially through the layers, with no looping or internal feedback, ensuring a unidirectional flow from the input to output. The typical FFNN structure includes three core layers: an input unit, an intermediate (hidden) processing stage, and a final output node. Each layer passes its processed values forward to the next. The last layer delivers the predicted class or outcome based on the input data. Model training involves repeatedly adjusting the connection strengths (weights) between neurons across layers. These updates are guided by optimization algorithms such as backpropagation and gradient descent. When properly optimized, the FFNN can uncover and learn complex, non-linear associations between input variables.

In an FFNN architecture, the total number of layers and neurons are key variables. Consequently, the performance of the FFNN is closely related to these parameters. We evaluated several FFNN architectures, finding the best performance with a single hidden layer and 10 neurons.

The choice of using a single hidden layer with ten neurons was guided by both empirical testing and the need to minimize overfitting due to the relatively small dataset. Although deeper networks were explored, they did not provide significant improvements in classification accuracy. Moreover, the simpler architecture ensured more stable training and generalization across 10-fold cross-validation. Thus, the selected FFNN structure effectively balances complexity and robustness.

The input layer includes as many neurons as there are features, while the output layer has two neurons, corresponding to the two classes (i.e., R and NR groups).

Two binary classification tasks are defined to evaluate the performance of the proposed CAD system: distinguishing R versus NR for SSRI and R versus NR for rTMS. The *k*-fold CV is a technique in machine learning used to avoid bias in the results. Here, it is employed to evaluate the performance of the proposed CAD system. In the *k*-fold CV technique, the input data are divided into *k* subsets, where the model is iteratively tested with one subset while being trained on the other subsets. Thus, all the subsets serve as the testing set once and as training sets k−1 times. In this study, a 10-fold CV strategy is applied during the training and testing of the FFNN architecture.

For both classification tasks, when a test EEG signal is input into the FFNN architecture for classification, the decision of the classifier is categorized into one of the following conditions:

- True Positive (TP): An R EEG signal is correctly classified as R.
- True Negative (TN): An NR EEG signal is correctly classified as NR.
- False Positive (FP): An NR EEG signal is incorrectly classified as R.
- False Negative (FN): An R EEG signal is incorrectly classified as NR.

Notably, since the task aims to detect R EEG signals, “true” and “positive” are defined for R labels and correct decisions, respectively. The classification ACC, SEN, and SPE are used to evaluate the performance of a CAD system. ACC represents reliability of the CAD system in correctly identifying R and NR signals. The SEN is defined as the CAD system’s ability to detect R signals (i.e., the target). Similarly, the SPE is defined as the CAD system’s ability to recognize NR signals (i.e., nontarget). These metrics are mathematically defined as follows:

(11) $$ACC=\frac{T_{TP}+T_{TN}}{T_{TP}+T_{TN}+T_{FP}+T_{FN}}\times 100$$

(12) $$SEN=\frac{T_{TP}}{T_{TP}+T_{FN}}\times 100$$

(13) $$SPE=\frac{T_{TN}}{T_{TN}+T_{FP}}\times 100$$

Here, $T_{TP}$, $T_{TN}$, $T_{FP}$, and $T_{FN}$ denote the total counts of TP, TN, FP, and FN after ten iterations of training and testing the CAD system during 10-fold CV strategy, respectively.

## 5. Results and Discussion

In this work, a new CAD system is developed to predict the outcomes of SSRI and rTMS therapies for depression disorder. Two different databases are used to evaluate the performance of the proposed CAD system. The first database is the Mumtaz database, which contains the EEG signals of 30 depression patients who are candidates for SSRI therapy. The second database is the Atieh Hospital database, which includes the EEG signals of 46 and 15 depression patients who are candidates for rTMS therapy. In this work, two binary classification tasks are defined to evaluate the performance of the proposed CAD system: R versus NR for SSRI therapy and R versus NR for rTMS therapy.

The EEG signals are split into 15-s segments. Since the sampling frequency in the Mumtaz database is 256 Hz, the EEG signals, after being split into 15-s intervals, have dimensions of 19×3840 (i.e., number of channels × number of samples). On the other hand, the sampling frequency of the Atieh Hospital database is 500 Hz, and as a result, the EEG signals in the Atieh Hospital database have dimensions of 19×7500.

Figure 2 shows the block diagram of the proposed CAD system for predicting the results of depression therapies. In the first step of the proposed CAD system, the EEG signals are de-noised using the MSPCA technique. Each channel of the EEG signal is subsequently plotted in 2D space using the APM technique. After that, the BPFSL technique is applied to the two-dimensional shapes and 32 features are extracted for each channel. Data fusion is then performed by concatenating the features of the 19 channels. This means that a total of 608 features are extracted from a 19-channel EEG signal (19 channels × 32 features = 608 features). In fact, the proposed CAD system reduces the dimension of the EEG data from 19×3840 in the Mumtaz database and 19×7500 in the Atieh Hospital database to 1×608. This represents a dimensionality reduction of approximately 99.17% in the Mumtaz database and 95.57% in the Atieh Hospital database. In other words, the proposed CAD system, by using the APM plot aligned with the BPFSL technique, extracts the pattern of the data while simultaneously reducing the data dimension.

**Figure 7:**
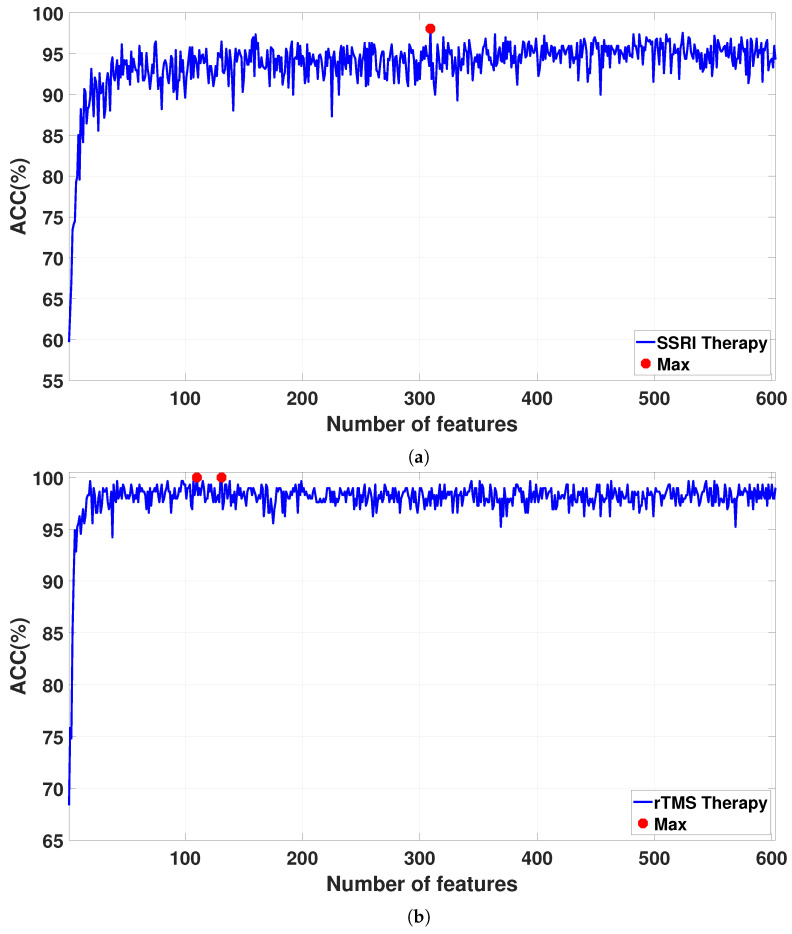

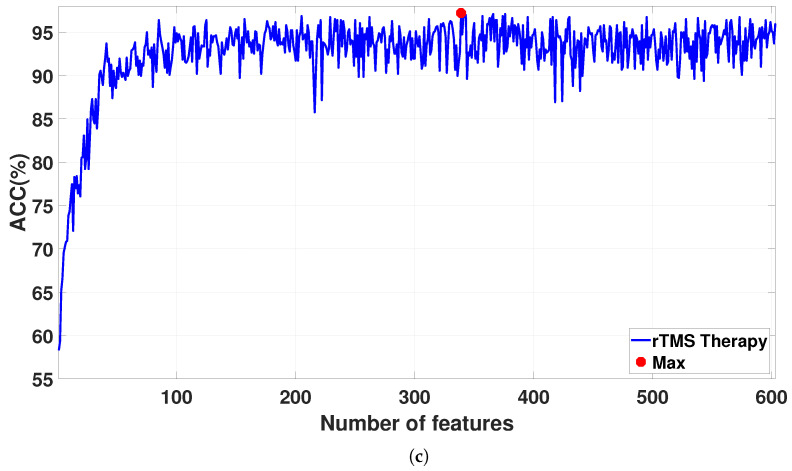
Classification ACC for selected features by the NCA algorithm in (**a**) SSRI therapy, (**b**) rTMS therapy for small Atieh Hospital database, and (**c**) rTMS therapy for the big Atieh Hospital database.

The features are sorted via the NCA algorithm on the basis of their weights. The FFNN architecture is used for feature classification. The features are iteratively increased from 1 to 608 based on their weights and are fed into the FFNN architecture via a 10-fold CV strategy for classification. Figure 7 shows the classification accuracies for the sorted features as the feature vector size increases from 1 to 608. The detailed results for the proposed CAD system are reported in Table 5. For SSRI therapy, the highest classification accuracy of 98.06% is achieved with 309 features (see Figure 7a). For rTMS therapy, a smaller Atieh Hospital database, the best classification accuracy of 100% is achieved with 110 and 131 features (see Figure 7b), while when the larger Atieh Hospital database is used, the best classification accuracy of 97.19% is achieved with 339 features (see Figure 7c).

**Figure 8:**
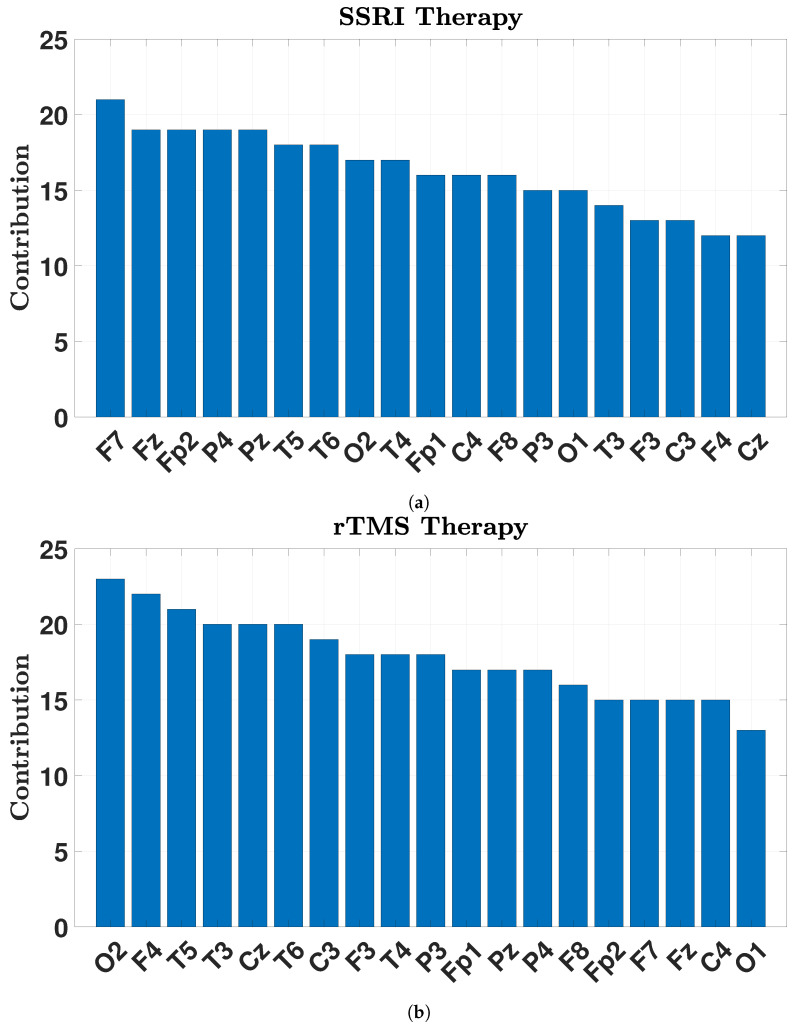
Contribution channels in SSRI (**a**) and rTMS (**b**) therapies.

The contribution of the channels based on the selected features by NCA is illustrated in Figure 8. From Figure 8, the most informative EEG channels for predicting SSRI therapy outcomes are identified as F7, Fz, Fp2, P4, and Pz, whereas for rTMS therapy, the most contributive channels are O2, F4, T5, T3, Cz, and T6. These spatial patterns reflect distinct neurophysiological mechanisms involved in each treatment modality.

For SSRI therapy, the key channels are located primarily in the prefrontal and parietal regions. F7 (left ventrolateral prefrontal cortex) and Fz (midline prefrontal/cingulate) are involved in emotional regulation, decision-making, and cognitive control—functions often impaired in depression [41]. Fp2, located over the right orbitofrontal cortex, plays a central role in reward processing and evaluating emotional salience, both of which are modulated by serotonergic systems [42]. P4 and Pz are associated parietal electrodes with the default mode network and attentional processes [43]. Their involvement suggests altered fronto-parietal dynamics in SSRI responders, potentially reflecting reorganization in emotion-regulation and self-referential networks, rather than direct causal modulation.

In contrast, the observed EEG patterns in rTMS-treated patients indicate broader network engagement across frontal, temporal, occipital, and central regions. F4 (right dorsolateral prefrontal cortex), the stimulation target, is critical for executive function and mood regulation [44,45]. O2 (right occipital lobe) is associated with visual processing and posterior alpha rhythms, possibly reflecting treatment-related shifts in arousal or sensory integration [46]. Temporal electrodes T5 (left posterior), T3 (left mid-temporal), and T6 (right posterior temporal) correspond to areas involved in emotional memory and limbic activity, frequently associated with depressive symptomatology [47]. Cz, located at the vertex over the sensorimotor cortex, may suggest engagement of motor-related circuits and cortico-striatal-thalamic loops, which have recently gained recognition in depression neurobiology.

Together, these results indicate that SSRI therapy primarily modulates prefrontal–parietal circuits associated with emotional regulation and self-referential thought, whereas rTMS therapy exerts its effects through broader cortical networks, including frontal, temporal, and occipital regions, reflecting its direct neuromodulatory influence and downstream connectivity changes.

To evaluate the contribution of each individual step of the proposed CAD system, the system is re-evaluated by dropping one specific step at a time, and the performance is checked. This means that the CAD system is rerun while excluding one specific step each time.

By dropping the MSPCA section of the CAD system, the performance decreases to 83.33%, 89.11%, and 76.16% in the Mumtaz, small Atieh Hospital, and big Atieh Hospital databases, respectively, resulting in decreases in performance of 14.73%, 10.89%, and 21.03%. It should be noted that by dropping the NCA section, as the feature selection technique, all 608 extracted features are fed into the FFNN architecture for classification. In this case, the classification ACC decreased from 98.06% to 95.59% for SSRI therapy. Thus, by using the NCA algorithm, the classification ACC is increased by 2.47%, while the number of features is reduced from 608 to 309 in SSRI therapy. Similarly, by dropping the NCA section in rTMS therapy, the classification ACC decreases from 100% to 98.97% for the small Atieh Hospital database and from 97.19% to 90.18% in the big Atieh Hospital database. Therefore, when the NCA algorithm is used, the classification ACC increases by 1.03% and 7.01%, while the number of features is decreased from 608 to 110 and from 608 to 339 in the small and big Atieh Hospital databases, respectively. These results highlight the impact of MSPCA and NCA algorithms as noise cancellation and feature selection techniques, respectively.

**Figure 9:**
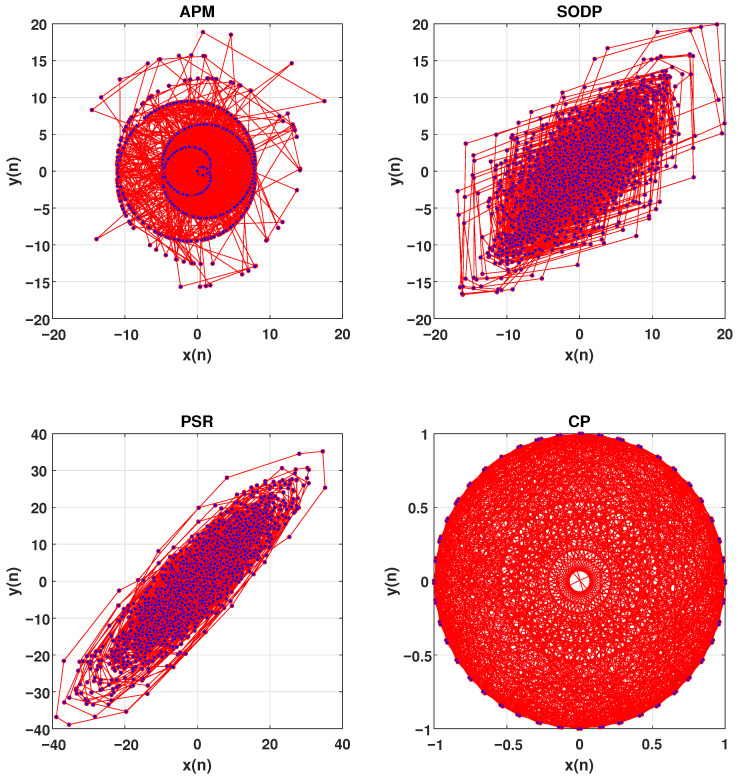
Comparison of the APM technique with SODP, PSR, and CP.

We plot the EEG signals in 2D space via the PSR [37], SODP [38], and CP techniques [39], instead of the APM technique, to demonstrate their ability to decode the nonlinear and complex behavior of EEG data. Figure 9 illustrates the 2D plot of a channel of an EEG signals using the APM technique, SODP, PSR, and CP. The classification ACC of 88.04% is obtained using PSR in SSRI therapy, which is approximately 10% lower than that of the APM technique. Similarly, when SODP is used, a classification ACC of 91.86% is achieved in SSRI therapy, which is approximately 6% lower than that of the APM technique. Using the CP technique, a classification ACC of 86.37% is obtained, which is again approximately 12% lower than that of the APM technique. This demonstrates the significant performance of the APM technique in decoding EEG data compared to traditional techniques.

SODP uses the differential operator to generate two time series, x(n) and y(n), from the signal [38]. On the other hand, CP uses sine and cosine functions to generate these two time series from the signal [39]. The SODP shows the variation in the EEG signal without capturing the chaotic nature of the EEG signals [38], whereas CP focuses on the nonlinear and chaotic behavior of the EEG signal by mimicking the patterns of sine and cosine [39]. The main weakness of CP is that it does not account for amplitude variations, which contain significant information about the signal’s behavior. In contrast, the APM technique uses the variation in amplitude aligned with sine and cosine to decode the nonlinearity of the EEG signals.

The performance of the proposed CAD system is re-evaluated via the KNN classifier as well. The classification ACC decreased from 98.06% to 93.48% with SSRI therapy. Likewise, it decreased from 100% and 97.19% to 95.29% and 92.73% in rTMS therapy using the small and big Atieh Hospital databases, respectively. This demonstrates the importance of a well-tuned FFNN architecture.

Providing easy-to-use software for a CAD system has a direct impact on its utilization by clinicians in healthcare environments [48]. Therefore, we developed software to visualize the proposed CAD system for the medical team. Since the project was conducted at the University of North Texas to predict the outcomes of depression therapy, we named the software UNT-DT. The UNT-DT utilizes multi-channel EEG signals to predict the outcomes of SSRI and rTMS therapy and recommends the best course of treatment for the patient.

**Figure 10:**
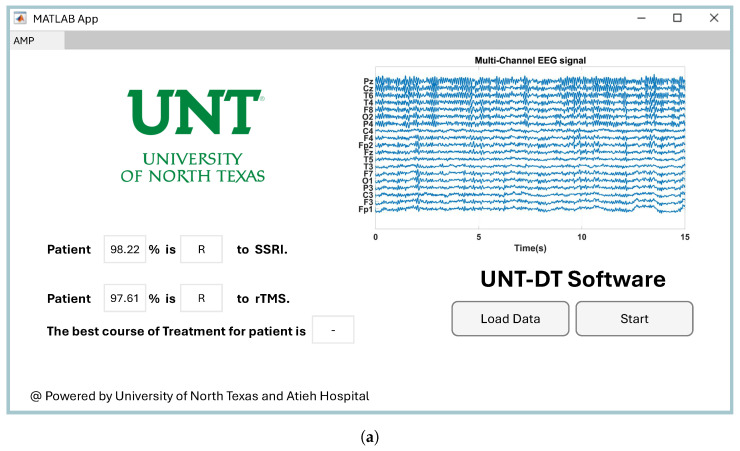

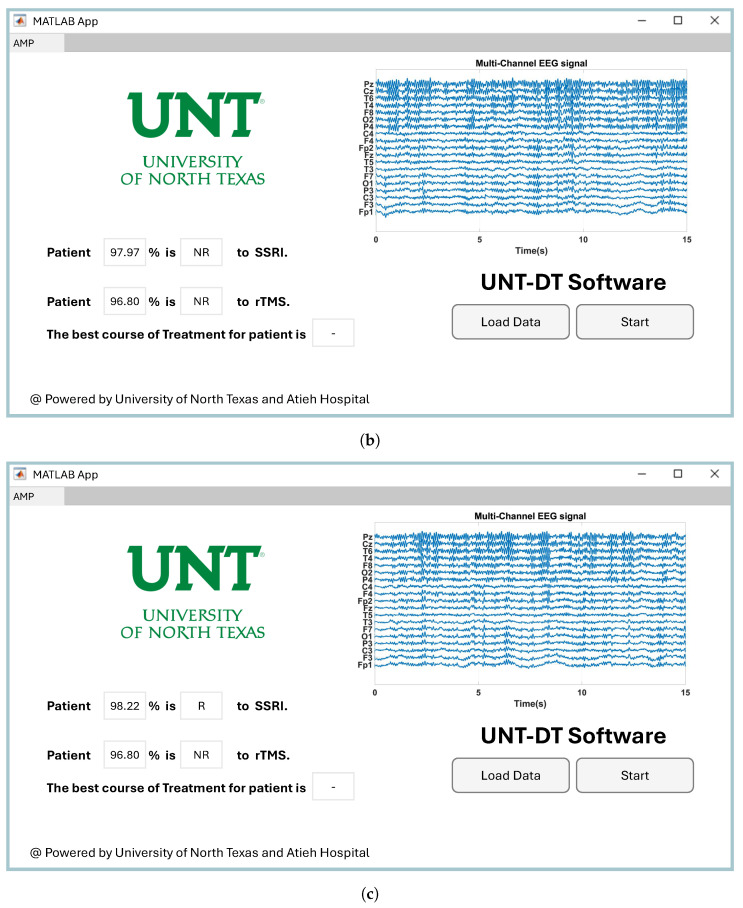

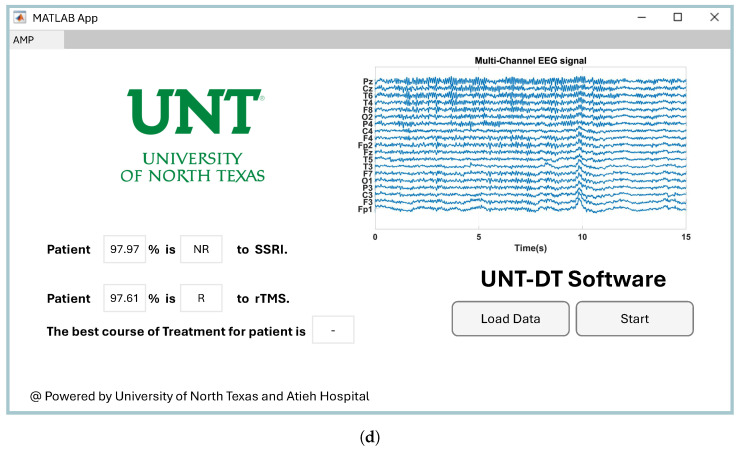
Visualization of four scenarios of the developed UNT-DT software: (**a**) Both SSRI and rTMS therapies result in R, (**b**) both therapies result in NR, (**c**) SSRI yields R while rTMS yields NR, and (**d**) SSRI yields NR while rTMS yields R.

The UNT-DT system categorizes patient outcomes into four distinct scenarios, depending on the combined results of rTMS and SSRI treatments. Two of these scenarios involve consistent responses to both therapies (either both positive or both negative), whereas the other two reflect differing outcomes—where one therapy is effective and the other is not. A visual representation of the UNT-DT application can be found in Figure 10.

Table 6 offers a comparison of the proposed CAD system’s effectiveness with that of the previous research that utilized the Mumtaz database for forecasting SSRI therapy results, and with recently released research on rTMS therapy employing both the small and big Atieh Hospital datasets. The proposed CAD system achieves perfect classification accuracies of 98.06%, 100%, and 97.19% for the Mumtaz, small Atieh Hospital, and large Atieh Hospital databases, respectively.

In [6], the EEG signals are split into several subbands which requires a complex time-frequency signal decomposition techniques. However, our proposed CAD system does not rely on any time-frequency technique before feature extraction and achieved a classification ACC of 98.06%, which is higher than the reported ACC of 91.60% in [6] for predicting the outcomes of SSRI therapy.

In [25], the continuous wavelet transform (CWT) was utilized to transform EEG signals into the time-frequency domain, which was subsequently expressed as images and incorporated into a transfer learning (TL) framework. This strategy obtained an accurate classification rate of 96.55%, which is 1.51% less than the accuracy obtained by our approach for predicting SSRI therapy results. The creation of image representations through continuous wavelet transform (CWT) is a far more complicated process than the direct projection of EEG signals into two-dimensional space utilising the amplitude polar map (APM) technique. Additionally, the proposed CAD framework uses a bandpass filter signal localisation (BPFSL) for feature extraction and implements a single-layer feedforward neural network (FFNN) for classification. In contrast, the methodology presented in [25] utilised a hybrid framework that integrates five transfer learning models: DenseNet121, Xception, VGG16, InceptionResNetV2, and MobileNetV2, which consist of 121, 36, 16, 164, and 53 layers, respectively, leading to a total of 390 layers. Thus, their methodology incorporates numerous convolutional and pooling operations, resulting in significant computational requirements. The proposed CAD system exhibits enhanced efficiency, attaining high accuracy while requiring considerably less computational power.

In [27], the feature map was extracted by applying Xception, VGG16, and DenseNet121 to the EEG signal as three pretrained TL architectures. The extracted feature map was then fed into a Bio-LSTM classifier. The proposed model in [27] requires significant computational resources for both feature extraction and classification, whereas our proposed CAD system extracts features via the APM technique aligned with BPFSL and classifies them using a single-layer FFNN architecture. As a result, our proposed CAD system is faster due to its simplicity, compared to the model in [27].

In a related study [22], a bidirectional long short-term memory (Bi-LSTM) classifier was coupled with four pretrained transfer learning architectures to analyse connectivity images. With 121, 66, 48, and 18 levels, the models used DenseNet121, EfficientNet-B0, Inception-v3, and ResNet18 to produce a deep learning framework with 253 layers. Although the model proposed in [22] reported a classification ACC of 98.33%, which is 0.25% higher than our proposed CAD system in detection of SSRI therapy results, generating images by measuring connectivity and aligning them with four successive TL architectures is more complex than our proposed CAD system. Our CAD system offers faster, simpler, and more straightforward computational steps.

In a related study [23], connection maps were constructed from the EEG channel relationship and went through three pretrained deep learning architectures: VGG16, Xception, and EfficientNetB0. These attributes were then supplied into a bidirectional long short-term memory (Bi-LSTM) network enriched with an attention procedure for classification. In addition, an optimal majority voting mechanism was applied to combine the results of the three designs. Using a leave-one-out cross-validation method, the model attained a classification accuracy of 99.32%. The classification ACC of our proposed CAD system is 97.19%, which is 2.15% lower than that of the proposed model in [23]. However, our CAD system has easier and fewer computational steps. Additionally, our proposed CAD system was evaluated via a 10-fold CV strategy, which provides stricter conditions than the leave-one-out CV strategy, making the reported results from our model more reliable and significant for real-world applications. In [26], CWT is employed to convert time series EEG signals into time-frequency images. These images were input into EfficientNetB0 and VGG16. Then, the output of these two pretrained TL architectures were integrated with a Bio-LSTM network aided with an attention mechanism for classification. They noted a 97.10% classification ACC using the EfficientNetB0-Bio-LSTM for predicting the results of rTMS therapy, which is 0.09% less than the accuracy attained by our suggested approach.

To predict rTMS treatment results from EEG data, three pretrained transfer learning models—VGG16, EfficientNetB0, and InceptionResNetV2—were integrated with a Bi-LSTM network in [28]. A weighted majority voting technique was used to build the ensemble model, and the differential evolution (DE) algorithm was used to optimise the weights. However, their technique obtained a classification accuracy of 98.51%, which is approximately 1.32% higher than our suggested system for rTMS outcome prediction, which was based on three transfer learning models, which increased architectural complexity when compared to our CAD framework.

In a different study [31], the connection matrices were constructed across four EEG rhythms and analyzed via five pretrained transfer learning methods architectures. The outputs of these models were pooled via a weighted majority voting system. This technique had a classification accuracy of 92.28% for predicting rTMS therapeutic results; however, our suggested CAD system surpassed it with a perfect accuracy of 100% on the same dataset.

The proposed CAD system extracts the complex and chaotic patterns of EEG data by plotting the EEG signals in a 2D space and computing the BPFSL for the shape. The best features are selected via NCA and fed into a single-layer FFNN architecture. In contrast, available methods used a variety of time-frequency and connectivity-based methodologies to first create feature matrices, which were subsequently combined with transfer learning models. Therefore, the proposed CAD system is faster and involves fewer computational steps compared with previous models, which require significant computational resources due to their complexity [6,22,23,25,26]. Additionally, for the first time in the literature, a CAD system has been developed to predict the outcomes of both SSRI and rTMS therapies for depression patients. The contributions and advantages of the proposed CAD system in the current work are as follows:

- Introduction of the amplitude polar map (APM) via a cutting-edge method for two-dimensionally representing the complex dynamics of EEG data.
- Proposal of the BPFSL feature to efficiently capture and define the nonlinear behaviour present in EEG recordings.
- Validation of the proposed CAD framework on two different EEG datasets with varied sampling rates, proving its flexibility and insensitivity to fluctuations in sampling frequency.
- While previous research has examined only one therapy, this work is the first to proposes a CAD system that can predict treatment results for both the SSRI and rTMS treatments in depression.
- Design of a computationally lightweight CAD framework that incorporates an efficient method for deriving features, leveraging the structural simplicity of APM and BPFSL, alongside a tuned feedforward neural network (FFNN) architecture comprising a single intermediate layer with ten neurons. This design avoids resource-intensive approaches such as connectivity-based [17,21,22] and time-frequency domain analyses [6,25], resulting in a fast, lightweight, and low-complexity implementation for EEG classification.
- In the proposed CAD system, the features are extracted by fusing the information from all EEG channels, meaning that it uses all the information in the EEG data without losing any information (i.e., no channels are dropped), while simultaneously reducing data redundancy.
- A dependable CAD framework was developed, and its performance was assessed through a 10-fold cross-validation approach to minimize potential evaluation bias.
- Development of a novel user-friendly, economical, and useful tool for forecasting the results of SSRI and rTMS therapy. Its MATLAB implementation facilitates portability and user-friendliness in clinical settings, aiding mental health professionals in making decisions about patient treatment.

Notably, clinical and demographic traits have shown some promise in predicting the results of the treatment. Patients with depression between the ages of 18 and 65 years were included in this study; the mean ages of the responder (R) and non-responder (NR) groups receiving SSRI therapy were 40.7 and 41.1 years, respectively (see Table 2). There was a modest difference in mean age between the NR and R groups, but it was not statistically significant (*p* > 0.05) [6]. Both the R and NR groups demonstrated equal gender representation, with an identical number of male and female participants (see Table 2) [6]. Remarkably, the R and NR groups’ respective pre-treatment BDI-II scores were 18.4 (±7.4) and 22.8 (±12.5) [6]. These results imply that NR patients had somewhat more severe symptoms than their R counterparts before treatment.

Demographic information and clinical characteristics of the Atieh Hospital rTMS database are provided in Table 4. The study involved 46 depression patients, equally divided into R and NR groups for rTMS therapy, with each group containing 23 patients. The mean age for the R group undergoing rTMS therapy is 30.87 years (±12.00), while for the NR group, it is 39.00 years (±14.16). While individuals in the NR group exhibited a higher average age compared to those in the R group, this age-related variation did not reach statistical significance (*p* = 0.052). In terms of gender distribution, both groups have a similar composition, with 8 males and 15 females in each group, and no significant gender difference between the groups (*p*-value = 0.90).

The pre-treatment BDI-II scores show a slight difference, with the R group scoring 32.5 (±9.3) and the NR group scoring 28.1 (±9.4), though this difference is not statistically significant (*p*-value = 0.08). However, the post-treatment BDI-II scores reveal a significant difference, with the R group showing a marked improvement (8.6±5.9) compared to the NR group (23.1±8.4), with a *p*-value of <0.001. Finally, the duration of depression shows no significant difference between the two groups, with the R group having a mean duration of 6.5 years (±8.2) and the NR group having a mean duration of 7.9 years (±7.8), with a *p*-value of 0.27.

In summary, the analysis revealed no meaningful statistical variation in demographic or clinical attributes between the Mumtaz and Atieh Hospital datasets. This suggests that the CAD model developed in this study is specifically tailored to distinguishing between responder (R) and non-responder (NR) groups. Variables such as age, BDI-II scores, gender, and other clinical indicators were found to have minimal impact on the classification performance.

While the proposed method demonstrated promising results in predicting therapy outcomes using EEG data, several limitations should be acknowledged. First, the dataset used in this study, although clinically relevant, was relatively limited in size, which may affect the generalizability of the findings. Second, the use of a private database may hinder reproducibility by other researchers. Third, the feature extraction process relies on predefined signal properties, which may not capture the full complexity of brain dynamics across all patients. To address these issues, future work will involve validating the model on larger and publicly available EEG datasets to ensure broader applicability. Additionally, we plan to explore deep learning-based end-to-end frameworks that learn feature representations automatically, potentially improving classification accuracy and interpretability. Cross-site validation and real-time deployment strategies will also be investigated to evaluate the model’s clinical utility.

Although gender information was available, the dataset was not balanced across male and female participants, and the sample size per gender group was insufficient for reliable subgroup analysis. This limits the ability to draw gender-specific conclusions from the current findings. Future studies should explore gender-stratified neural response patterns to improve personalized treatment prediction.

## 6. Conclusions

Accurately forecasting how patients respond to depression therapies remains a major obstacle in clinical psychiatry. Among the widely accepted interventions are selective serotonin reuptake inhibitors (SSRIs) and repetitive transcranial magnetic stimulation (rTMS), both of which are commonly prescribed based on the severity of the condition. Although approved by the FDA, these treatments often show varied effectiveness across individuals. In this work, a CAD system is developed to predict the outcomes of depression therapies and suggest the best course of treatment. The proposed CAD system uses a novel EEG signal illustration and feature engineering approach, referred to as the APM and BPFSL methods, respectively. The illustration is on the basis of the nonlinear fusion of the single channel of EEG with itself.

In the first step of the proposed CAD system, each channel of the EEG signal is plotted into a 2D space via the APM technique to represent the brain’s complexity. Subsequently, the BPFSL method is employed as a feature extraction technique to decode the complexity of the APM shape in the 2D space. Afterward, data fusion is performed by concatenating the features from all channels. The optimal set of features is identified using the NCA method and subsequently input into an FFNN architecture comprising one layer with 10 neurones. The performance of the proposed CAD system was rigorously examined across three different datasets: the Mumtaz dataset and two datasets acquired from Atieh Hospital, aiming to predict treatment outcomes for SSRI and rTMS therapies, respectively. The results obtained with a 10-fold CV approach suggest that a classification ACC of 98.06% was used to estimate the effects of SSRI treatment. Similarly, classification ACCs of 100% and 97.19% are reached for predicting the results of rTMS therapy utilising the small and large Atieh Hospital datasets, respectively.

Importantly, the analysis revealed that different sets of EEG channels play a dominant role in predicting responses to each therapy. SSRI outcomes were most strongly associated with prefrontal and parietal channels (F7, Fz, Fp2, P4, and Pz), which are related to emotional regulation, reward processing, and executive function. In contrast, rTMS outcomes were linked to a broader set of regions including frontal, temporal, occipital, and central channels (O2, F4, T5, T3, Cz, and T6), indicating widespread network engagement through neuromodulation. These findings provide valuable neurophysiological insights that could help tailor treatment selection in clinical settings.

Future validation of the proposed CAD framework will investigate its utility across broader spectrum of neuromodulatory therapies. These include traditional approaches like electroconvulsive therapy (ECT), transcranial magnetic and direct current stimulation (TMS, tDCS), and implantable technologies such as vagus nerve stimulation (VNS) and deep brain stimulation (DBS). We also aim to extend our framework to emerging modalities—including magnetic seizure therapy (MST), focused ultrasound stimulation, cranial electrical stimulation, and integrative interventions combining ketamine administration with mindfulness- or yoga-based treatments.

## Figures and Tables

**Table 2:** Demographic details of the Mumtaz dataset.

	R	NR
**Gender (Male & Female)**	8 & 8	9 & 9
**Average Age (Mean ± Standard Deviation)**	40.7 ± 13	41.1 ± 12.5
**BDI-II Before SSRI Therapy (Mean ± Standard Deviation)**	18.4 ± 7.4	22.8 ± 12.5
**BDI-II After SSRI Therapy (Mean ± Standard Deviation)**	9.1 ± 6.3	22.1 ± 3.3

**Table 3:** Summary of BDI-II scores for responders (R) and non-responders (NR) in the Small Atieh Hospital dataset.

	R	NR
**Number of Patients**	9	6
**BDI-II Before rTMS (Mean ± SD)**	27.56 ± 8.19	33.83 ± 12.08
**BDI-II After rTMS (Mean ± SD)**	7.44 ± 4.81	22.33 ± 10.59
**Improvement (%) (Mean ± SD)**	73.20 ± 13.57	33.06 ± 11.14

**Table 4:** Comparison of demographic and clinical characteristics between R and NR to rTMS in the Big Atieh Hospital dataset.

Characteristic	R	NR	*p*-Value
**Sample Size**	23	23	-
**Age (Mean ± Standard Deviation)**	30.87 ± 12.00	39.00 ± 14.16	0.052
**Gender (Male/Female)**	8/15	8/15	0.90
**BDI Score Before Therapy**	32.5 ± 9.3	28.1 ± 9.4	0.08
**BDI Score Before Therapy**	8.6 ± 5.9	23.1 ± 8.4	<0.001
**Length of Depressive Episode (Years)**	6.5 ± 8.2	7.9 ± 7.8	0.27

**Table 5:** The effectiveness of the suggested CAD system in forecasting the results of depression treatments.

Used Database	Therapy	ACC (%)	SEN (%)	SPE (%)	Number of Features
Mumtaz	SSRI	98.06	98.22	97.97	309
Small Atieh Hospital	rTMS	100	100	100	110
		100	100	100	131
Big Atieh Hospital	rTMS	97.19	97.61	96.80	339

**Table 6:** Performance comparison between the proposed CAD system and existing approaches using the Mumtaz and Atieh Hospital datasets.

Reference	Therapy Type	Dataset Used	Methodology	ACC (%)	SEN (%)	SPE (%)
[6]	SSRI	Mumtaz	Short-Time Fourier Transform + Empirical Mode Decomposition + Wavelet Transform + Logistic Regression	91.60	90.00	90.00
[25]	SSRI	Mumtaz	Continuous Wavelet Transform + Transfer Learning + Ensemble Voting	96.55	96.01	96.95
[27]	SSRI	Mumtaz	EEG Feature Extraction + Ensemble Transfer Learning + Bidirectional Long Short-Term Memory Network	98.84	97.80	99.60
[22]	SSRI	Mumtaz	Connectivity Maps + Ensemble Transfer Learning + Bidirectional Long Short-Term Memory Network	98.33	-	-
[31]	rTMS	Small Atieh Hospital	Transfer Learning + Differential Evolution Optimization	92.28	-	-
[23]	rTMS	Big Atieh Hospital	Connectivity Images + Ensemble Transfer Learning	99.32	-	98.34
[26]	rTMS	Big Atieh Hospital	Continuous Wavelet Transform Images + EfficientNetB0 + Bidirectional Long Short-Term Memory Network	97.10	97.30	97.00
[28]	rTMS	Big Atieh Hospital	Ensemble Transfer Learning + Differential Evolution Optimization	98.51	98.64	98.36
Proposed CAD system	SSRI	Mumtaz	Multiscale Principal Component Analysis + Amplitude Polar Mapping + Band-Pass Filtered Signal Localization + Neighborhood Component Analysis + Feedforward Neural Network	98.06	98.22	97.97
	rTMS	Small Atieh Hospital		100	100	100
	rTMS	Big Atieh Hospital		97.19	97.61	96.80

## Data Availability

The EEG data used in this study are publicly available on Figshare at https://figshare.com/articles/dataset/EEG_Data_New/4244171 (accessed on 1 January 2025).
